# Contextualizing stigma in Parkinson's disease research

**DOI:** 10.1016/j.clinsp.2024.100425

**Published:** 2024-06-28

**Authors:** Tomás de la Rosa, Fúlvio Alexandre Scorza

**Affiliations:** a Neuroscience Department, Universidad de Cádiz, Cádiz, Spain; b Instituto de Investigación e Innovación Biomédica de Cádiz (INiBICA), Cádiz, Spain; c Centro de Investigación Biomédica en Red en Salud Mental (CIBERSAM), Instituto de Salud Carlos III, Madrid, Spain; d Neurology Department, Escola Paulista de Medicina Universidade Federal de São Paulo, São Paulo, SP, Brazil

Recent research in Parkinson's Disease (PD) has underscored the necessity of examining stigma within a broader framework that includes social, cultural, and political dimensions.^1^^,^^2^ This recognition emphasizes some limitations of traditional medical perspectives and calls for an expanded focus on factors such as social discrimination, socioeconomic status, past traumas, access to specialized care, and caregiving disparities.^3^ The incorporation of diverse and underrepresented populations should help us to better understand and address the multifaceted challenges faced by individuals with PD in their respective contexts.^4^ The call for attention to social, cultural, and political disparities in stigma aligns with the overarching goal of understanding the intricate nature of the issue and constitutes a global trend in medical care.^5^^,^^6^

Stigma, generally, can be delineated as a characteristic that implies the devaluation of the afflicted, categorizing them as “bad, weak, or dangerous”, thereby diminishing their identity from that of a complete, ordinary individual to one marked as tainted.^7^ These individuals are sometimes depicted as lacking autonomy and self-control, which impedes their constitution as subjects capable of political and social action. In the context of PD, stigma manifests through multiple dimensions, starting with the visible symptoms, such as tremors or dyskinesia, together with the progressive loss of functionality and autonomy.^2^ This stigma can reach around 60% of PD patients,^8^ affecting how individuals with PD are viewed and treated by others. Increased dependency on caregivers and medical interventions challenges societal values of autonomy and self-sufficiency, further entrenching stigmatizing attitudes. Beyond these motor and physical symptoms, the PD burden also encompasses psychosocial challenges that can detrimentally affect patient well-being and self-esteem. A prominent concern pertains to the prevalent stigma encountered by PD patients on account of their medical condition, arising from the interplay between individuals and their social environment (Maffoni et al., 2017). The notion of dependency not only impacts the self-perception of the patients but also influences how they are perceived by themselves, and within their social networks and community.^4^ This internalized stigma can lead to feelings of shame, low self-esteem, and social withdrawal, further impairing the individual's quality of life and hindering their engagement. There is also an intersectional aspect to the stigma associated with PD Factors such as age, gender, socioeconomic status, and cultural background play significant roles in shaping the experience of stigma.^1^ Older adults, who constitute the majority of PD patients, may already face age-related discrimination. This discrimination is exacerbated by the symptoms and prognosis associated with PD, often placing them in a delicate position regarding their quality of life and mortality.^9^

While PD serves as a tool for anamnesis in neuropsychiatry, it's essential to recognize its deep entwinement with social, cultural, and political factors, impacting the construction of this stigma. As previously highlighted, researchers are increasingly advocating for this recognition as they endeavor to alleviate the repercussions of stigma in patients. However, they often do this by examining disease variables that have little to do with the sociocultural matrix surrounding the patient. For example, a recent review by Karacan and colleagues,^1^ observed that most PD studies concentrated on the correlation between stigma and clinical attributes while paying little attention to the potential sociocultural factors that could help us understand the role of culture and society on stigma. It was observed in this review that motor impairment and treatment were linked to significantly heightened experiences of stigma. Such correlations between clinical symptoms and stigma are a common theme in stigma research,^4^ often leading to the logical inference that ameliorating these symptoms is the key to effectively diminishing stigma among PD patients. Implying the possibility of identifying further biomedical variables as potential predictors. While evidence suggests a connection between improved motor symptoms and reduced stigma in PD patients, focusing solely on symptom and functional capacity places the stigma on the bodies of patients,^10^ neglecting its fundamental sociocultural dimension. This concern is often acknowledged in the introduction or discussion sections of studies, with authors emphasizing the significance of contextualizing the stigma phenomenon. Nevertheless, their examination of variables continues to predominantly center on physical conditions and symptoms, essentially revolving around bodily states. This narrow focus overlooks the broader socio-cultural and psychological dimensions of stigma, limiting the understanding of how various intersecting factors, such as age, gender, socioeconomic status, and cultural background, contribute to the lived experiences of individuals with PD. Consequently, there is a need for more comprehensive research approaches that integrate these multifaceted aspects to fully grasp the complexities of stigma in PD patients.

Clinicians and researchers operate within a determinate social and political space, which influences the biomedical care they provide. Beyond addressing the motor and non-motor symptoms impacting patient well-being, an expanded consideration of factors surrounding PD is needed. This broader perspective aims to redefine the understanding of PD and formulate responses that reflect a nuanced appreciation of patients' experiences. If stigma is acknowledged as primarily a social issue, the focus should transition from biomedical variables to an exploration of factors intricately linked with social discrimination and stigma, encompassing socioeconomic status, prior trauma, access to specialists, and caregiving. Addressing these dimensions necessitates a collaborative effort involving healthcare professionals, and researchers in medical and social sciences, alongside family members and patients.

## Authors’ contributions

Both T.R. and F.S. contributed equally to the conceptualization, writing, and reviewing of the present manuscript.

## Funding

This study was supported by the following grants: FAPESP (Fundação de Amparo à Pesquisa do Estado de São Paulo), CNPq (Conselho Nacional de Desenvolvimento Científico e Tecnológico) and Ministério de Universidades. The authors report no other sources of funding or conflicts of interest.

## Conflicts of interest

The authors declare that the research was conducted in the absence of any commercial or financial relationships that could be construed as a potential conflict of interest.
